# Clinically relevant body composition methods for obese pediatric patients

**DOI:** 10.1186/s12887-019-1454-2

**Published:** 2019-03-21

**Authors:** Alexandra Kreissl, Anselm Jorda, Katharina Truschner, Gabriele Skacel, Susanne Greber-Platzer

**Affiliations:** Department of Pediatrics and Adolescent Medicine, Division of Pediatric Pulmology, Allergology and Endocrinology, Medical University of Vienna, Waehringer Guertel 18-20, 1090 Vienna, Austria

**Keywords:** Bioelectrical impedance analysis, Body composition, Pediatric obesity, Body fat percentage, Fat mass

## Abstract

**Background:**

There is no gold standard in body composition measurement in pediatric patients with obesity. Therefore, the aim of this study was to investigate if there are any differences between two bioelectrical impedance analysis techniques performed in children and adolescents with obesity.

**Methods:**

Data were collected at the Department of Pediatrics and Adolescent Medicine in Vienna from September 2015 to May 2017. Body composition measurement was performed with TANITA scale and BIA-BIACORPUS.

**Results:**

In total, 38 children and adolescents (age: 10–18 years, BMI: 25–54 kg/m^2^) were included. Boys had significantly increased fat free mass (TANITA *p* = 0.019, BIA *p* = 0.003), total body water (TANITA *p* = 0.020, BIA *p* = 0.005), and basal metabolic rate (TANITA *p* = 0.002, BIA *p* = 0.029). Girls had significantly increased body fat percentage with BIA (BIA *p* = 0.001). No significant gender differences of core abdominal area have been determined. TANITA overestimated body fat percentage (*p* < 0.001), fat mass (*p* = 0.002), and basal metabolic rate (*p* < 0.001) compared to BIA. TANITA underestimated fat free mass (*p* = 0.002) in comparison to BIA. The Bland Altman plot demonstrated a low agreement between the body composition methods.

**Conclusions:**

Low agreement between TANITA scale and BIA-BIACORPUS has been observed. Body composition measurement should always be performed by the same devices to obtain comparable results. At clinical routine due to its feasibility, safety, and efficiency, bioelectrical impedance analysis is appropriate for obese pediatric patients.

**Trial registration:**

ClinicalTrials NCT02545764. Registered 10 September 2015.

## What is already known on this topic

1. Obesity has tripled in the last forty years and represents already a health issue in childhood.
2. The body composition differs essentially between children and adults.
3. There is a lack of knowledge of body composition in pediatric patients with obesity.

## What this paper adds

1. This study provides data on the comparison of the two most clinically relevant body composition methods in pediatric obese patients.
2. Low agreement between TANITA scale and BIA-BIACORPUS has been observed, TANITA scale overestimated body fat percentage, fat mass, and basal metabolic rate in comparison to BIA.
3. Body composition measurement should always be performed with the same body composition devices to obtain comparable results.

## Background

Worldwide reports reveal that obesity has tripled in the last 40 years [1]. In 2016, 41 million children younger than 5 years and 340 million children and adolescents aged between five to nineteen years, were overweight or obese [1]. Overweight and obesity represent already a health issue in childhood. Obesity in early childhood is associated with an increased risk of obesity in adulthood [1]. Therefore, prevention is already needed at a young age.

Obesity is a heterogeneous disease and is characterized by abnormal or excessive fat accumulation. Body fat percentage (BFP) is strongly associated with the risk of several chronic diseases and a massive increased fat mass leads to obesity defined as abnormal or excessive fat accumulation [2]. The body composition (BC) differs essentially between children and adults [3]. In addition, gender, age, health conditions, and ethnical background have an impact on body composition [4, 5]. However, it’s accurate measurement is of utmost importance. The body mass index (BMI) does not differentiate between fat mass (FM) and fat free mass (FFM) [6]. Different body composition measurement techniques exist and range from underwater weighing (densitometry), dual energy x-ray absorptiometry (DEXA), magnetic resonance imaging (MRI) to bioelectrical impedance analysis (BIA). Most of these methods either expose the patient to radiation, are time consuming, inconvenient and expensive. Therefore, the non-invasive bioelectrical impedance analysis measurement appears to be a useful and feasible tool, especially for young pediatric populations. This device is fast in the measurement, inexpensive compared to other techniques, gives reliable measurements of body composition with minimal intra and inter-observer variability and is reproducible with < 1% error on repeated measurements [7].

There is a lack of knowledge of body composition in pediatric patients with obesity. Moreover, no data are available on the comparison of two clinically relevant body composition methods for children. The aim of this study was to investigate the differences between two bioelectrical impedance analysis techniques performed in children and adolescents with obesity. Furthermore, we analyzed if gender has an impact on the body composition in children and adolescents.

## Methods

### Study design, patient recruitment and allocation

Data were acquired within the Children’s KNEEs study and sample size calculation was based on 48 patients including a 20% drop out rate [8]. All children and adolescents aged between 10 and 18 years, with a BMI percentile defined as > 97 percentile according to Kromeyer-Hauschild [9] and who were patients at the outpatient clinic of obesity, lipometabolic disorder and nutritional medicine, Department of Pediatrics and Adolescent Medicine, Medical University of Vienna were included in this study from September 2015 to May 2017. Patients with obesity associated syndromes, chronic joint diseases, osteoarthritis surgery and neuro-motor diseases were excluded. All parents and their children were informed by a study team member at the outpatient clinic and written informed consent was obtained from all participants and their legal representatives. The study was approved by the local Ethics-Committee of the Medical University Vienna (MUV, EC Nr: 1445/2013).

### Anthropometric measurements

Anthropometric measurements were conducted in patients wearing only light clothes and were performed by the same study team members. Body weight (kg) was measured in sitting position, through a calibrated scale (SECA 959, Seca Gmbh & Co, Hamburg, Germany) and was recorded within 0.1 kg precision. The next anthropometric measurements were recorded to the nearest 0.1 cm. Body height (cm) measurement was performed of the maximum distance from the floor to the highest point on the head, when the subject was facing directly ahead in a standing position. Shoes were off, feet together, and arms by the sides, heels, buttocks and upper back were also in contact with the wall when the measurement was performed with a calibrated stadiometer (SECA 264, Seca Gmbh & Co, Hamburg, Germany). The body mass index (BMI) (kg/m^2^) was calculated as body weight divided by body height squared using the formula (weight in kg)/(height in m)^2^. Waist circumference (WC) (cm) was measured at the midpoint between the lower margin of the last palpable rib and the top of the iliac crest [10]. Waist circumference was the point of minimal waist measured with an anthropometric non-elastic tape (PRYM Group, Stolberg, Germany) [11]. The WC is an indicator of central body fat. Hip circumference was measured around the widest portion of the buttocks. During measuring tape was parallel to the floor. The waist-to-hip ratio (WHR) (cm) is calculated as waist measurement (cm) divided by hip circumference (cm) [12]. The waist-to-height ratio (WHtR) is calculated as waist circumference (cm) divided by height (cm). Abdominal circumference (cm) was measured as the maximum circumference between the lower margin of the last palpable rib and the top of the iliac crest with a non-elastic tape (PRYM Group, Stolberg, Germany). Mid upper-arm circumference (MUAC) (cm) was measured with a non-elastic tape (PRYM Group, Stolberg, Germany) in a standing position on the relaxed (and to the side free hanging) upper right arm. MUAC was defined as the upper arm circumference at the 50% distance between the acromion and the olecranon.

### Body composition (BC)

Body composition was performed with two different methods, the TANITA scale and BIA-BIACORPUS device, which are both based on bioelectrical impedance analysis (BIA). The measurement was always performed by the same study team members. The patients were always measured at the same point of time in the morning, with an empty bladder, and with calibrated scales, wearing light underwear in standing position for TANITA and immediately afterwards in a lying position for the BIA-BIACORPUS analysis. To assure that variances through different methods of determination were minimized, all study team members in the Department of Pediatrics and Adolescent Medicine were trained study personnel.

#### TANITA

For analysis the patients’ age, gender, and body height was typed in without decimal number. In addition, the physical activity status of the patients was entered for each measurement, by choosing body type “standard” or “athletic”. For all patients the “standard” type was selected. The analysis was conducted in a standing position bare feet, thighs as well as arms were not touching each other. Body height was entered, body weight was measured and BMI was calculated. The TANITA scale (Type BC-418MA, Tokyo, Japan) is a bioelectrical impedance analysis device. This segmental body composition analyser measures body fat percentage (BFP, %), fat mass (FM, kg), fat free mass (FFM, kg), total body water (TBW, kg), basal metabolic rate (BMR, kcal) and predicted muscle mass (MM) derived from dual energy X-ray absorptiometry (DEXA) method using bioelectrical impedance analysis (BIA). The body fat percentage indicates the proportion of fat to the total body weight. Fat mass represents the actual weight of fat within the body. Fat free mass consists of muscle, bone, tissue and water in the body. Total body water exhibits the total amount of fluid in the patient’s measured body. The basal metabolic rate reveals the daily minimum energy requirement at rest to function appropriately. The predicted muscle mass comprises the bone-free lean tissue mass. The TANITA device measured with an eight-electrode bioelectrical impedance analysis, which was supplied from the tips of the toes of both feet and from the fingertips of both hands, with a measurement frequency of 50 kHz. The five different impedance measurements of right leg, left leg, right arm, left arm and the trunk were quantified [13].

#### BIA

The BIA-BIACORPUS (BIACORPUS RX 4000, Medical Healthcare GmbH, Karlsruhe, Germany) was used for assessing the body composition. The device is a non-invasive and fast tool to examine the body fat percentage, fat mass, fat free mass, total body water, body cell mass (BCM), extracellular mass (ECM), and basal metabolic rate with a measurement frequency of 50 kHz. Body cell mass comprises metabolically active tissues and extracellular mass is the amount of metabolically inactive tissue of the measured individual. The patient was lying horizontally for 5 min without any movements, in order to ensure a homogeneous distribution of the liquid. The arms were located laterally of the body, and the legs were slightly apart, as the limbs and the body did not touch each other. Eight adhesive electrodes were attached on the body, two on each foot and hand [14].

### Statistical methods

Statistical analyses were performed using the software Statistical Package for Social Science (SPSS Inc., Chicago, IL, version 24.0). *P*-values of < 0.05 were considered statistically significant. Results are expressed as mean and standard deviation (SD), or median and range, and percentage. The unpaired t-test was used to determine gender differences. The paired t-test was used for the comparison of body composition methods. Values for body composition of BIA and TANITA were compared by the Pearson correlation coefficient for correlation and the Bland-Altman plot [15] to assess agreement between the two methods and 95% limits of agreement were calculated as the mean difference ± 1.96 SD.

## Results

### Study population

In total, 38 patients, 14 (37%) female and 24 (63%) male children and adolescents with obesity, were included in the study. Study participants were 13 years (=median, range: 10–18 years) old with a body height of 163 cm (=median, range: 121–192 cm) and with a BMI of 35 kg/m^2^ (=median, range: 25–54 kg/m^2^).

### Baseline characteristics

Descriptive information of the study population groups is given in Table 1.

**Table 1:** Patients Demographic Data

Characteristics	All Patients
n	38
Male (%)	24 (63%)
Age (years)	13.3 ± 2.3
SBP (mmHg)	125 ± 12
DBP (mmHg)	69 ± 9
Pulse (1/min)	84 ± 14
Height (cm)	162.5 ± 12.8
Weight (kg)	95.8 ± 30.1
BMI (kg/m^2^)	34.4 ± 6.8
Abdominal-C (cm)	110.3 ± 13.8
Hip-C (cm)	110.2 ± 14.6
Waist-C (cm)	98.3 ± 12.2
MUAC (cm)	36.7 ± 5.1
WHtR	0.61 ± 0.08
WHR	0.89 ± 0.06

Results are given as mean ± SD or as number of subjects (%). *SBP* systolic blood pressure, *DBP* diastolic blood pressure, *BMI* body mass index, *C* circumference, *MUAC* mid-upper arm circumference, *WHtR* Waist-to-height ratio is calculated as waist circumference (cm) divided by height (cm), *WHR* Waist-to-hip ratio is calculated as waist measurement (cm) divided by hip circumference (cm)

### Gender differences

Gender differences have been observed in the body composition measured with TANITA and BIA device (Table 2). Male patients had a significantly increased fat free mass (TANITA *p* = 0.019, BIA *p* = 0.003), total body water (TANITA *p* = 0.020, BIA *p* = 0.005), and basal metabolic rate (TANITA *p* = 0.002, BIA *p* = 0.029). Female patients had more body fat percentage, which was significantly increased with BIA (*p* = 0.001).

**Table 2:** Gender Differences in the Body Composition measured with TANITA and BIA

Characteristics	Male (*N* = 24)	Female (*N* = 14)	*p*-value	Male (*N* = 24)	Female (*N* = 14)	*p*-value
	TANITA			BIA		
BFP (%)	39.7 ± 7.9	42.5 ± 7.0	0.279	35.5 ± 4.7	42.0 ± 6.5	**0.001**
FM (kg)	40.3 ± 18.3	37.9 ± 15.4	0.677	35.8 ± 13.4	37.3 ± 14.8	0.743
FFM (kg)	58.7 ± 14.5	48.1 ± 9.5	**0.019**	63.2 ± 15.7	48.6 ± 9.6	**0.003**
TBW (kg)	43.0 ± 10.6	35.2 ± 7.0	**0.020**	45.2 ± 12.1	34.6 ± 7.1	**0.005**
BMR (kcal)	2133 ± 407	1721 ± 259	**0.002**	1698 ± 211	1516 ± 282	**0.029**
Segmental Analysis measured with TANITA						
	Right leg			Left leg		
BFP (%)	42.0 ± 6.7	46.8 ± 6.3	**0.038**	42.9 ± 7.0	47.1 ± 6.0	0.065
FM (kg)	9.2 ± 4.5	8.1 ± 3.3	0.431	9.3 ± 4.9	7.9 ± 3.2	0.357
FFM (kg)	12.0 ± 3.5	8.7 ± 2.1	**0.003**	11.7 ± 3.4	8.5 ± 2.1	**0.003**
MM (kg)	11.4 ± 3.3	8.2 ± 2.0	**0.002**	10.7 ± 3.4	8.0 ± 2.0	**0.012**
	Right arm			Left arm		
BFP (%)	42.6 ± 8.3	49.5 ± 6.6	**0.012**	47.6 ± 9.7	54.5 ± 7.9	**0.030**
FM (kg)	2.3 ± 1.2	2.4 ± 1.1	0.917	3.0 ± 2.1	3.2 ± 1.6	0.805
FFM (kg)	3.0 ± 0.9	2.2 ± 0.6	**0.005**	3.0 ± 0.9	2.4 ± 0.6	**0.020**
MM (kg)	2.8 ± 0.8	2.1 ± 0.5	**0.006**	2.9 ± 0.8	2.2 ± 0.6	**0.014**

Results are given as mean ± SD. Bold values indicate significant difference at the *p* < 0.05 level. Comparisons were made using independent samples t-test. *BIA* bioelectrical impedance analysis, *BFP* body fat percentage, *FM* fat mass, *FFM* fat free mass, *TBW* total body water, *BMR* basal metabolic rate, *MM* muscle mass

With BIA measurement it has been indicated that male patients had an increased extracellular mass (*p* = 0.011) and an increased body cell mass (*p* = 0.002).

With TANITA significant gender differences have been observed in segmental analysis of right and left leg as well as in right and left arm (Table 2). Girls had an increased body fat percentage in the right leg as well as in right and left arm (*p* < 0.05). Male individuals had significantly more fat free mass and muscle mass in all four body parts (*p* < 0.05). No significant gender differences of core abdominal area have been determined.

### Comparison between BIA and TANITA

The measured TANITA body weight was used and entered for the BIA measurement to guarantee the comparability of the two methods. The comparison of both methods demonstrated that the results of TANITA and BIA device differed significantly from each other (*p* < 0.05) (Table 3). Data showed that TANITA scale overestimated body fat percentage (*p* < 0.001), fat mass (*p* = 0.002), and basal metabolic rate (*p* < 0.001) compared to BIA. TANITA underestimated fat free mass (*p* = 0.002) in comparison to BIA.

**Table 3:** Methods Comparison of TANITA vs. BIA

Characteristics	TANITA (*N* = 38)	BIA (*N* = 38)	*p*-value
BFP (%)	40.8 ± 7.6	37.9 ± 6.2	**0.000**
FM (kg)	39.4 ± 17.1	36.4 ± 13.8	**0.002**
FFM (kg)	54.8 ± 13.7	57.8 ± 15.4	**0.002**
TBW (kg)	40.1 ± 10.1	41.3 ± 11.7	0.114
BMR (kcal)	1982 ± 409	1631 ± 252	**0.000**

Results are given as mean ± SD. Bold values indicate significant differences at the p < 0.05 level. Comparisons of the two methods were made using paired sample t-test. *BIA* bioelectrical impedance analysis, *BFP* body fat percentage, *FM* fat mass, *FFM* fat free mass, *TBW* total body water, *BMR* basal metabolic rate

**Fig. 1:**
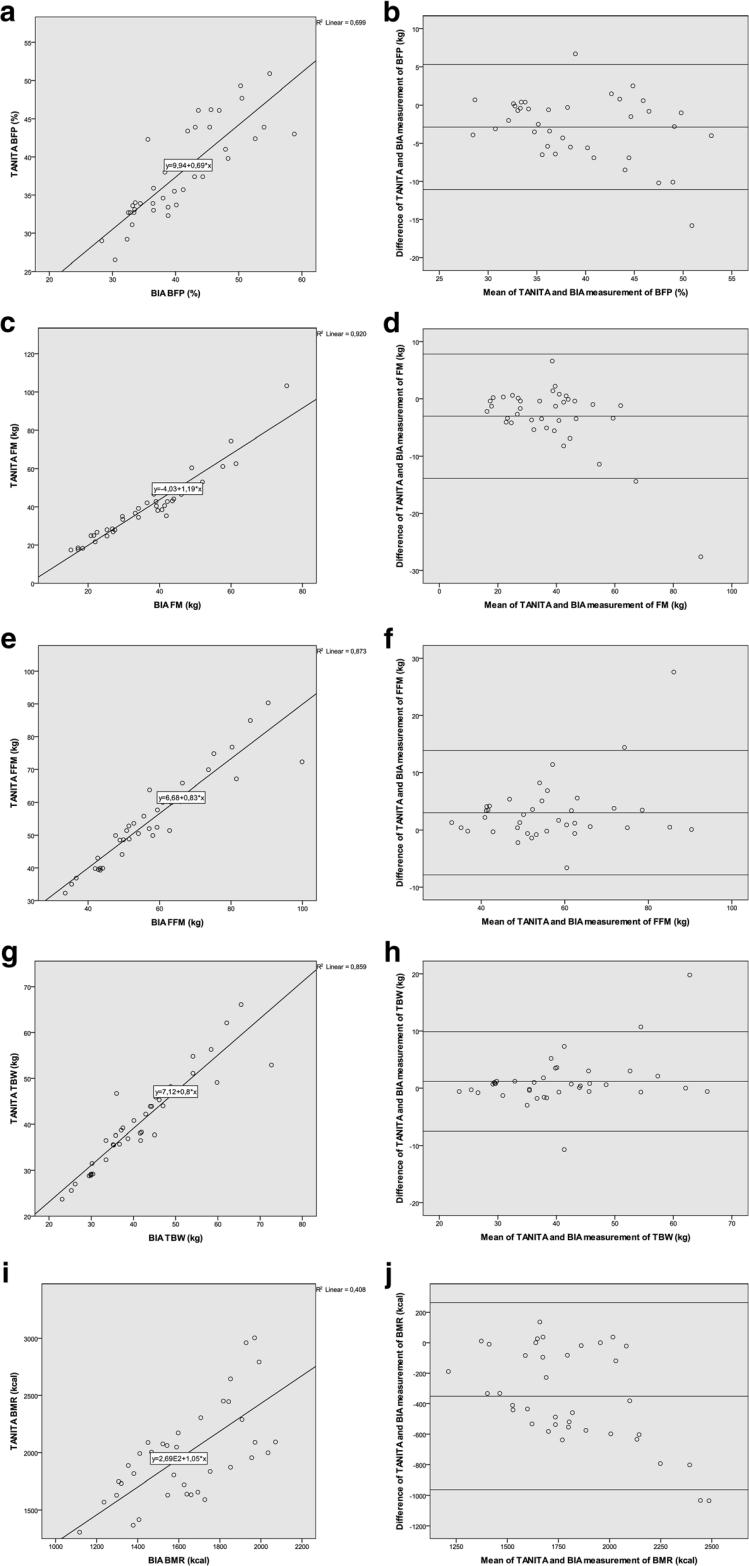
Relationship of BIA to TANITA and Bland Altman plots. Pearson correlation coefficient between BIA and TANITA method of body fat percentage (**a**), fat mass (**c**), fat free mass (**e**), total body water (**g**) and basal metabolic rate (**i**). Bland Altman plot of BIA and TANITA for body fat percentage (**b**), fat mass (**d**), fat free mass (**f**), total body water (**h**), and for basal metabolic rate (**j**). Mean difference and limits of agreement are displayed as reference lines. BIA = bioelectrical impedance analysis, BFP = body fat percentage, FM = fat mass, FFM = fat-free mass, TBW = total body water, BMR = basal metabolic rate

The Pearson correlation and the Bland-Altman plot is displayed in Fig. 1. The Pearson correlation showed a significant positive correlation between BIA and TANITA methods for body fat percentage (Pearson r = 0.836; *p* < 0.001, *n* = 38), fat mass (Pearson r = 0.959; *p* < 0.001, n = 38), fat free mass (Pearson r  =  0.934; *p*  <  0.001, n  =  38), total body water (Pearson r  =  0.927; *p*  <  0.001, n  =  38), and basal metabolic rate (Pearson r  =  0.646; p  <  0.001, n  =  38). In Fig. 1: a, c, e, g, i: line represents linear regression of data in body fat percentage (y  =  9.94x + 0.069; r^2^  =  0.699), fat mass (y  =  4.03x + 1.19; r^2^  =  0.920), fat free mass (y  =  6.68x + 0.83; r^2^  =  0.873), total body water (y  =  7.12x + 0.8; r^2^  =  0.859) and basal metabolic rate (y  =  2.69x + 1.05; r^2^  =  0.408).

The upper and lower margin was defined by mean ± 1.96 SD of the difference [16]. Therefore, 95% of all samples are included in the margin (Fig. 1: b, d, f, h, j). The Bland Altman plot shows the best agreement with total body water (mean: 1.2 kg, 95% limits of agreement: − 7.5 to 9.9 kg) compared to body fat percentage (mean: − 2.9 kg, 95% limits of agreement: − 11.1 kg to 5.3 kg), fat mass (mean: − 3.0 kg, 95% limits of agreement: − 13.9 kg to 7.8 kg), fat free mass (mean: − 3.0 kg, 95% limits of agreement: − 7.8 to 13.9 kg), and basal metabolic rate (mean: 350.5 kcal, 95% limits of agreement: − 962.4 to 261.5 kcal) between BIA and TANITA.

## Discussion

In the present study, body composition of a young population with obesity was evaluated by using two methods, namely TANITA and BIA-BIACORPUS devices. We observed low agreement between the two body composition methods and Bland-Altman postulates the non acceptable level of agreement between the two methods.

Significant differences between both body composition methods were detected. The Pearson correlation coefficient is commonly used for measuring the association between two methods. Results might be misleading and therefore Bland Altman plot has been used to determine the limits of agreement. The Bland Altman plots demonstrated a low agreement between the body composition methods. An upper and lower margin of more than ±5 kg can be defined as clinically relevant. The outcomes demonstrated that fat mass, fat free mass and total body water were exceeding this threshold. Moreover, the upper and lower margin of body fat percentage was more than ±5%, and basal metabolic rate was more than ±250 kcal. TANITA overestimated body fat percentage, fat mass and basal metabolic rate. The higher the total body fat percentage and fat mass was, the higher the deviation between the measurements was observed. The outcomes indicate that body composition should always be performed by the same body composition device to obtain comparable results.

We have observed gender differences in regard to the body composition. Male individuals had with TANITA and BIA an increased muscle mass, which results in an increased energy burning. This in coherence with our findings of an accelerated and higher basal metabolic rate and was displayed in a decreased body fat percentage and increased fat free mass. Male individuals usually have increased total body water, as they have an increased muscle mass [17]. Those outcomes are consistent with our results measured with TANITA and BIA. In girls, body fat percentage and fat mass were increased in comparison to boys, which is in line with the literature. One main factor is puberty induced fat deposition in hips, thighs and growing breast tissue in the girls [18]. Body fat percentage is a main factor to determine obesity. The outcomes established that body fat percentage for obesity in girls was over their age recommended cut-off levels of ≥35% body fat percentage and in boys over the cut-off ≥25% body fat percentage [19]. The fat distribution has an impact on health, which can be determined with waist circumference. An increased health risk is defined by a waist circumference in women > 88 cm and for men > 102 cm [20]. For children and adolescents-specific cut-off values are not available. However, our results indicated that girls as well as boys were above the announced cut-off values for waist circumference and have therefore an increased health risk. Intra-abdominal, also called visceral body fat, is located in the core abdominal area and is correlated with an increased health risk [1]. No significant gender differences in abdominal fat have been noticed. However, patients had an increased android fat distribution, which is related with an increased cardiovascular and metabolic risk as well as insulin resistance [21].

Recent data explored that in Austrian children and adolescents aged 4 to 19 years, 18% of boys and 12% of girls were overweight and 5% of boys and 3% of girls were obese [22]. Hence, overweight and obesity is already an issue in Austria and prevention is needed. An increased body mass in particular increased body fat percentage is strongly associated with the risk of several chronic diseases [1]. During growth the body composition changes [3].

The percent body fat increases about 11% from infancy to 6 months of age. Then, body fat percentage decreases (males 11%, girls 7%) from 6 months up to the age of 10 years [4]. Especially in patients with obesity, the body composition has to be monitored.

There is no gold standard in the body composition measurement in a pediatric study population with obesity. However, the TANITA scale and BIA-BIACORPUS are non-invasive, fast and easy in the measurement, and highly accepted by children and adolescents. Bioelectrical impedance analysis does not require exposure to radioactivity or submersion in water, and therefore it is a practical measure of body composition, especially in the clinical routine with children. Bioelectrical impedance analysis devices, like TANITA, are frequently used in a young population. Several studies conducted in children and adolescents have shown a good level of accuracy in comparison to reference body composition methods [23–26].

However, other studies revealed a low level of accuracy [27–30], which might be caused by differences in used BIA devices, reference methods and study populations in regard to ethnicity and age.

Detailed information about the body composition in children and adolescents with obesity evaluated with two different body composition methods have not been evaluated so far in Austria. This study explores the body composition measurement differences of two methods used at a clinical setting in such a pediatric study population with obesity. Body composition should always be measured with the same device to obtain comparable results.

A limitation of this study is the lack of other analysis techniques such as DEXA, MRI or air displacement plethysmography [31]. However, those methods cannot be used within the clinical routine in pediatric obese patients. DEXA, which is often announced to be a gold standard in body composition measurement, is in pediatric patients not feasible as this method involves radiation exposure and the procedure takes up to 20 min and patients should not move within the measurement. Moreover, trained radiology personnel is required and the measurement is expensive. There is no gold standard in body composition measurement in pediatric patients with obesity. However, at clinical routine due to its feasibility, safety, and efficiency, bioimpedance analysis is the most often used body composition method, especially performed in pediatric patients.

## Conclusions

In conclusion, data suggest a low agreement between the TANITA scale and the BIA-BIACORPUS. TANITA overestimated body fat percentage, fat mass, and basal metabolic rate in comparison to BIA. Body composition measurement should always be performed by the same devices to obtain comparable results. Nonetheless, at clinical routine due to its feasibility, safety and efficiency, bioimpedance analysis seems appropriate for obese pediatric patients.

## Abbreviations

BC: Body composition
BCM: Body cell mass
BFP: Body fat percentage
BIA: Bioelectrical impedance analysis
BMI: Body mass index
BMR: Basal metabolic rate
C: Circumference
DBP: Diastolic blood pressure
DEXA: Dual-energy x-ray absorptiometry
ECM: Extracellular mass
FFM: Fat free mass
FM: Fat mass
MM: Muscle mass
MRI: Magnetic resonance imaging
MUAC: Mid-upper arm circumference
SBP: Systolic blood pressure
SD: Standard deviation
SPSS: Statistical Package for Social Science
TBW: Total body water
WHR: Wait-to-hip ratio
WHtR: Waist-to-height ratio

## References

[1] WHO. Fact sheet obesity and overweight. http://www.who.int/mediacentre/factsheets/fs311/en/. Accessed 22 Apr 2018.

[2] Sharma AM, Chetty VT (2005). Obesity, hypertension and insulin resistance. Acta Diabetol.

[3] Kyle UG, Earthman CP, Pichard C, Coss-Bu JA (2015). Body composition during growth in children: limitations and perspectives of bioelectrical impedance analysis. Eur J Clin Nutr.

[4] Fomon SJ, Haschke F, Ziegler EE, Nelson SE (1982). Body composition of reference children from birth to age 10 years. Am J Clin Nutr.

[5] Talma H, Chinapaw MJ, Bakker B, HiraSing RA, Terwee CB, Altenburg TM (2013). Bioelectrical impedance analysis to estimate body composition in children and adolescents: a systematic review and evidence appraisal of validity, responsiveness, reliability and measurement error. Obes Rev.

[6] Ellis KJ, Shypailo RJ, Abrams SA, Wong WW (2000). The reference child and adolescent models of body composition. A contemporary comparison. Ann N Y Acad Sci.

[7] Diaz EO, Villar J, Immink M, Gonzales T (1989). Bioimpedance or anthropometry?. Eur J Clin Nutr.

[8] Horsak B, Artner D, Baca A, Pobatschnig B, Greber-Platzer S, Nehrer S (2015). The effects of a strength and neuromuscular exercise programme for the lower extremity on knee load, pain and function in obese children and adolescents: study protocol for a randomised controlled trial. Trials..

[9] Kromeyer-Hauschild K, Wabitsch M, Kunze D, Geller F, Geiß HC, Hesse V (2001). Perzentile für den Body-mass-Index für das Kindes- und Jugend-alter unter Heranziehung verschiedener deutscher Stichproben. Monatsschrift Kinderheilkunde.

[10] WHO (2000). Obesity: preventing and managing the global epidemic.

[11] Ross R, Berentzen T, Bradshaw AJ, Janssen I, Kahn HS, Katzmarzyk PT (2008). Does the relationship between waist circumference, morbidity and mortality depend on measurement protocol for waist circumference?. Obes Rev.

[12] WHO. Waist Circumference and Waist-Hip Ratio Report of a WHO Expert Consultation.Geneva; 2008: 47.

[13] TANITA Corporation. Body composition analyzer BC-418 instructional manual. Tokyo, Japan:1–23.

[14] Medical Healthcare GmbH. Bedienungsanleitung Biacorpus RX 4000. Germany: 2013:1–19.

[15] Bland JM, Altman DG (1986). Statistical methods for assessing agreement between two methods of clinical measurement. Lancet..

[16] Bland JM, Altman DG (1995). Comparing methods of measurement: why plotting difference against standard method is misleading. Lancet..

[17] Butte NF, Hopkinson JM, Wong WW, Smith EO, Ellis KJ (2000). Body composition during the first 2 years of life: an updated reference. Pediatr Res.

[18] Leonard MB, Elmi A, Mostoufi-Moab S, Shults J, Burnham JM, Thayu M (2010). Effects of sex, race, and puberty on cortical bone and the functional muscle bone unit in children, adolescents, and young adults. J Clin Endocrinol Metab.

[19] McCarthy HD, Cole TJ, Fry T, Jebb SA, Prentice AM (2006). Body fat reference curves for children. Int J Obes.

[20] Kopelman PG (2000). Obesity as a medical problem. Nature..

[21] Snijder MB, Visser M, Dekker JM, Goodpaster BH, Harris TB, Kritchevsky SB (2005). Low subcutaneous thigh fat is a risk factor for unfavourable glucose and lipid levels, independently of high abdominal fat. Health ABC Study Diabetologia.

[22] Mayer M, Gleiss A, Hausler G, Borkenstein M, Kapelari K, Kostl G (2015). Weight and body mass index (BMI): current data for Austrian boys and girls aged 4 to under 19 years. Ann Hum Biol.

[23] Iacopino L, Andreoli A, Innocente I, Minisci C, Di Giorgio G, Anastasi A (2003). Use of foot-to-foot bioelectrical impedance analysis in children. Acta Diabetol.

[24] Sung RY, Lau P, Yu CW, Lam PK, Nelson EA (2001). Measurement of body fat using leg to leg bioimpedance. Arch Dis Child.

[25] Lim JS, Hwang JS, Lee JA, Kim DH, Park KD, Jeong JS (2009). Cross-calibration of multi-frequency bioelectrical impedance analysis with eight-point tactile electrodes and dual-energy X-ray absorptiometry for assessment of body composition in healthy children aged 6-18 years. Pediatr Int.

[26] Yu OK, Rhee YK, Park TS, Cha YS (2010). Comparisons of obesity assessments in over-weight elementary students using anthropometry, BIA, CT and DEXA. Nutr Res Pract.

[27] Azcona C, Koek N, Fruhbeck G (2006). Fat mass by air-displacement plethysmography and impedance in obese/non-obese children and adolescents. Int J Pediatr Obes.

[28] Goldfield GS, Cloutier P, Mallory R, Prud'homme D, Parker T, Doucet E (2006). Validity of foot-to-foot bioelectrical impedance analysis in overweight and obese children and parents. J Sport Med Phys Fit.

[29] Lazzer S, Boirie Y, Meyer M, Vermorel M (2003). Evaluation of two foot-to-foot bioelectrical impedance analysers to assess body composition in overweight and obese adolescents. Br J Nutr.

[30] Sluyter JD, Schaaf D, Scragg RK, Plank LD (2010). Prediction of fatness by standing 8-electrode bioimpedance: a multiethnic adolescent population. Obesity..

[31] Jebb SA, Elia M (1993). Techniques for the measurement of body composition: a practical guide. Int J Obes Relat Metab Disord.

